# Genome/transcriptome collection of plethora of economically important, previously unexplored organisms from India and abroad

**DOI:** 10.1016/j.dib.2019.104099

**Published:** 2019-06-05

**Authors:** Arijit Panda, Narendrakumar M. Chaudhari, Mayuri Mukherjee, Samrat Ghosh, Aditya Narayan Sarangi, C. Mathu Malar, Shashi Kant, Diya Sen, Abhishek Das, Subhadeep Das, Deeksha Singh, Asharani Prusty, Sucheta Tripathy

**Affiliations:** a Academy of Scientific and Innovative Research (AcSIR), CSIR–Indian Institute of Chemical Biology, 4, Raja S.C. Mullick Road, Jadavpur, Kolkata 700032, India; b Structural Biology and Bioinformatics Division Department, CSIR–Indian Institute of Chemical Biology, 4, Raja S.C. Mullick Road, Jadavpur, Kolkata 700032, India

**Keywords:** Annotation, Genome, Transcriptome

## Abstract

Genome and transcriptome sequencing data are extremely useful resources for researchers in carrying out biological experiments that involves cloning and characterizing genes. We are presenting here genome sequence data from different clades of life including photosynthetic prokaryotes; oomycetes pathogens; probiotic bacteria; endophytic yeasts and filamentous fungus and pathogenic protozoa *Leishmania donovani*. In addition, we are also presenting paired control and treated stress response transcriptomes of Cyanobacteria growing in extreme conditions. The Cyanobacterial species that are included in this dataset were isolated from extreme conditions including desiccated monuments, hot springs and saline archipelagos. The probiotic *Lactobacillus paracasei* was isolated from Indian sub-continent. The Kala azar causing protozoan *Leishmania donovani*, whose early infectious stage is also included in this dataset. The endophyte *Arthrinium malaysianum* was isolated as a contaminant has significant bio-remediation property. Our collaborators have isolated endophyte *Rhodotorula mucilaginosa* JGTA1 from Jaduguda mines, West Bengal, India infested with Uranium. Our collaborators have isolated a heterozygous diploid oomycetes pathogen, *Phytophthora ramorum* causing sudden oak death in CA, USA coast is also part of the data. These dataset presents a unique heterogeneous collection from various sources that are analyzed using “Genome Annotator Light (GAL): A Docker-based package for genome analysis and visualization” (Panda et al., 2019) and are presented in a web site automatically created by GAL at http://www.eumicrobedb.org/cglab.

**Table:** Specifications Table

Subject area	Biology, Genetics
More specific subject area	Genomics, Computational Genomics, Transcriptomics
Type of data	Genome sequences (text),Transcriptomic raw data, Alignment files in SAM/BAM format, Database (online data and graphics)
How data was acquired	Next Generation DNA and RNA Sequencing
Data format	Filtered, annotated and analyzed complete genomes, assemblies and raw transcriptomic sequences
Experimental factors	Collected organisms from various sources such as monuments, hot springs, saline archipelagos, hosts with pathogens, endophytes etc.
Experimental features	The genome and transcriptomic sequencing of microbes are carried out by second and third generation sequencing platforms.
Data source location	India; California, USA
Data accessibility	The collection of analyzed genomes of various microbes is publically accessible at following url: http://www.eumicrobedb.org/cglab This resource is created after a collection of genomes sequenced in our laboratory at CSIR-Indian Institute of Chemical Biology, Kolkata, India and in collaborative labs. Newly sequenced additional genomes will be added to the same repository from time to time in future. The details of the data with their accession numbers are provided in Table 1.

**Table:** 

**Value of the data** 1. The importance of this data collection lies with the economic significance of the organisms sequenced. Here, mostly extremophile Cyanobacteria and fungi isolated from diverse sources are included. There is one fungi like organism which is a pathogen on Oak plants was isolated from CA coast is also part of this resource. 2. Another aspect of this collection is the availability of not just the sequence data but the analyzed collection of large datasets of genomes and their annotations with comparative genomics in comparative genomics visualization in browser tracks. 3. The genomes sequenced hereby are mostly from Indian subcontinent, which unleash the hidden biodiversity of highly diverse Indian lands, oceans and monuments. 4. Genome sequenced pathogens which destroy commercially important plants may prove to be of immense importance to the researchers and economists of the related countries. 5. The probiotic and immune-modulatory activities of some microbes can be explored using this data collection where genomes from probiotic strains e.g. *Lactobacillus casei* Lbs2 isolated from gut of Indian individual were sequenced and assembled. 6. Economically important yeasts genome is also present in this collection, e.g. it includes genome of the plant growth promoting yeast *Rhodotorula mucilaginosa* JGTA-S1, found in *Typha angustifolia*, a macrophyte growing in wetland near to Uranium-mine in Jaduguda, India. 7. Also transcriptomic data of some crucial microbes are provided to study expression profiles. 8. Overall, these data may create new possibilities of collaboration in given context i.e. genomics and transcriptomic aspects of previously unexplored organisms

## 1 Data

The data collection includes sequenced and analyzed genomes of cyanobacteria, fungi, yeast and oomycetes from our laboratory and from the laboratories of our collaborators. This data collection offers a repository of genomic information of economically or environmentally important microorganisms. The collection currently includes only genomics data and transcriptomics data for some Cyanobacterial genomes and fungi under a paired experiment [Table 1]. The collection does not currently include proteomic or metabolomic data.

**Table 1:** List of selected microbes sequenced and analyzed in the data collection with their data accession and publications.

Sr. No.	Taxonomy	Full Name in NCBI	Data Type	NCBI BioSample	NCBI BioProject	NCBI Accession	Data Source	Citation
1	Cyanobacteria	*Halomicronema excentricum* str. Lakshadweep	Genomic	SAMN09787254	PRJNA485276	QVFV00000000.1	Marine archipelagos from the Lakshadeep islands	UP
2	Cyanobacteria	*Hassallia byssoidea* VB512170	Genomic	SAMN03174155	PRJNA266752	JTCM00000000	Desiccated monuments, of eastern India	[2]
3	Cyanobacteria	*Lyngbya confervoides* BDU141951	Genomic	SAMN03217274	PRJNA268230	JTHE00000000	Marine Cyanobacteria from Southern India	[3]
4	Cyanobacteria	*Mastigocladus laminosus* UU774	Genomic	SAMN05942614	PRJNA350610	MNPM00000000	Hot springs of eastern India	UP
5	Cyanobacteria	*Scytonema millei* VB511283	Genomic	SAMN03200207	PRJNA267760	JTJC00000000	Desiccated monuments, of eastern India	[4]
6	Cyanobacteria	*Scytonema tolypothrichoides* VB-61278	Genomic	SAMN03274355	PRJNA271448	JXCA00000000	Desiccated monuments, of eastern India	[5]
7	Cyanobacteria	*Tolypothrix bouteillei* VB521301	Genomic	SAMN02697214	PRJNA242379	JHEG00000000	Desiccated monuments, of eastern India	[6]
8	Cyanobacteria	*Tolypothrix campylonemoides* VB511288	Genomic	SAMN03274356	PRJNA271449	JXCB00000000	Desiccated monuments, of eastern India	[7]
9	Cyanobacteria	*Westiellopsis prolifica* IICB1	Genomic	SAMN06473105	PRJNA377809	NAPS00000000	Fresh water Cyanobacteria from culture collections of India	UP
10	Firmicutes	*Lactobacillus paracasei* Lbs2	Genomic	SAMN02910029	PRJNA255080	JPKN00000000	Isolated from the guts of healthy humans from Northern India.	[8]
11	Fungi	*Rhodotorula mucilaginosa* JGTA-S1	Genomic	SAMN07313544	PRJNA393004	PEFX00000000	Endophytes isolated from Jaduguda mine, eastern India.	[9]
12	Leishmania	*Leishmania donovani* strain:MHOM/IN/1983/AG83(early passage)	Genomic	SAMN04145217	PRJNA297706	GCA_001989975.1 (36 chromosomes)	Leishmania pathogens isolated from diseased rodents from India and the cultures represents early passage (<5 passages).	[10]
13	Oomycetes	*Phytophthora ramorum isolate:CDFA1418886*	Genomic	SAMN08537704	PRJNA434169	PUHL00000000	This is a virulent pathogen on Oak trees in Southern CA, USA. These organisms are fungi like but are distinctly different from them. PacBio sequencing was done on them to improve the assembly.	[11], [12]
14	Cyanobacteria	*Halomicronema excentricum* str. Lakshadweep	Trascriptomics	SAMN09787254	PRJNA485276	Submitted and Processing	Control and organisms grown under acid stress at pH 4.5.	UP
15	Cyanobacteria	*Mastigocladus laminosus* UU774	Trascriptomics	SAMN05942614	PRJNA350610	Submitted and Processing	Cyanobacteria grown under heat stress and control conditions.	UP
16	Cyanobacteria	*Scytonema tolypothrichoides* VB-61278	Trascriptomics	SAMN03274355	PRJNA271448	Submitted and Processing	Whole transcriptome	UP
17	Cyanobacteria	*Tolypothrix campylonemoides* VB511288	Trascriptomics	SAMN03274356	PRJNA271449	Submitted and Processing	Whole transcriptome	UP
18	Cyanobacteria	*Tolypothrix bouteillei* VB521301	Trascriptomics	SAMN02697214	PRJNA242379	Submitted and Processing	Whole transcriptome	UP

Note: UP; unpublished.

## 2 Experimental design, materials and methods

Table 1 lists the current set of complete genomes and transcriptomes from diverse sources sequenced or processed at our lab or in collaboration.

## 3 Other useful links to related resources

• **EumicrobeDBLite:** web resource for publically available Oomycete genomes (http://eumicrobedb.org) [13].
• **BGA Genomics:** a blue-green algae genome database. (http://bgagenomics.iicb.res.in/).
• **Genome Annotator Lite (GAL):** A standalone package for analysis and visualization of genomes. (https://hub.docker.com/r/cglabiicb/gal/) [1].
